# Cellular Injury of Cardiomyocytes during Hepatocyte Growth Factor Gene Transfection with Ultrasound-Triggered Bubble Liposome Destruction

**DOI:** 10.1155/2011/453619

**Published:** 2010-12-09

**Authors:** Kazuo Komamura, Rie Tatsumi, Yuko Tsujita-Kuroda, Takatoshi Onoe, Kunio Matsumoto, Toshikazu Nakamura, Jun-ichi Miyazaki, Takeshi Horio, Masaru Sugimachi

**Affiliations:** ^1^Department of Cardiovascular Dynamics, Research Institute, National Cerebral and Cardiovascular Center, 5-7-1 Fujishiro-dai,Suita, Osaka 565-8565, Japan; ^2^Department of Nursing Science, Taisei Gakuin University, Sakai 587-8555, Japan; ^3^Division of Molecular Regenerative Medicine, Department of Biochemistry and Molecular Biology, Osaka University Graduate School of Medicine, Suita 565-0871, Japan; ^4^Division of Stem Cell Regulation Research, G6, Osaka University School of Medicine, Suita 565-0871, Japan; ^5^Division of Hypertension, National Cerebral and Cardiovascular Center, Suita 565-8565, Japan

## Abstract

We transfected naked HGF plasmid DNA into cultured cardiomyocytes using a sonoporation method consisting of ultrasound-triggered bubble liposome destruction. We examined the effects on transfection efficiency of three concentrations of bubble liposome (1 × 10^6^, 1 × 10^7^, 1 × 10^8^/mL), three concentrations of HGF DNA (60, 120, 180 *μ*g/mL), two insonification times (30, 60 sec), and three incubation times (15, 60, 120 min). We found that low concentrations of bubble liposome and low concentrations of DNA provided the largest amount of the HGF protein expression by the sonoporated cardiomyocytes. Variation of insonification and incubation times did not affect the amount of product. Following insonification, cardiomyocytes showed cellular injury, as determined by a dye exclusion test. The extent of injury was most severe with the highest concentration of bubble liposome. In conclusion, there are some trade-offs between gene transfection efficiency and cellular injury using ultrasound-triggered bubble liposome destruction as a method for gene transfection.

## 1. Introduction

Ultrasound-triggered bubble liposome destruction (sonoporation) has been proposed as a safe nonviral means of gene therapy that can target many different cells or tissues. In the field of cardiovascular medicine, this method may have significant potential for the introduction of therapeutic genes directly into vascular cells or cardiomyocytes [1, 2]. Sonoporation can only be clinically effective if the dose-effect relationship between the amount of bubble liposome and transfection efficiency is first established. However, few reports have already examined this dose-effect relationship and the safety of the procedure [3].

Transfection efficiency in sonoporation depends on various conditions including type of microbubble, mode of ultrasound, frequency of ultrasound, intensity of acoustic pressure, concentration of microbubble, dose of DNA, duration of insonification, incubation time of cell with DNA, repeat count of insonification, type of targeted cell, and other physicochemical conditions like temperature and carbon dioxide concentration, [3]. Greenleaf et al. reported that ultrasound acoustic pressure, DNA concentration, and repeat count of insonification correlated with transfection rate [4]. Teupe et al. demonstrated that duration of insonification did not affect transfection rate [5]. Then, Chen et al. showed that transfection rate reached plateau when DNA concentration was increased [6].

Greenleaf et al. also showed that transfection rate peaked and fell off according to the change in liposome concentration [4]. They thought it might be derived from cellular toxicity of large amount of liposome. Li et al. reported that cell viability decreased along with the increase in microbubble concentration [1]. Guo et al. demonstrated that cell viability decreased with the increase in duration of insonification [7]. Suzuki et al. and Li et al. showed that cell viability decreased with the increase in ultrasound acoustic pressure [8, 9]. 

On the basis of those previous findings, we planned to examine the effects of amount of plasmid DNA, liposome concentration, duration of insonification, repeat count of insonification, and time of incubation with liposome, cell, and DNA on transfection rate, which was measured by means of HGF protein release into culture medium.

## 2. Materials and Methods

### 2.1. Cell Culture

Primary cultures of neonatal ventricular myocytes were prepared as described previously [10]. Briefly, apical halves of cardiac ventricles from 1-day-old Wistar rats were separated, minced, and dispersed with 0.1% collagenase type II (Worthington Biochemical Corp., Freehold, NJ). Myocytes were segregated from nonmyocytes using a discontinuous Percoll gradient (Sigma Chemical Co., Inc., St. Louis, MO). After centrifugation, the upper layer consisted of a mixed population of nonmyocyte cell types and the lower layer consisted almost exclusively of cardiac myocytes. After the myocytes had been incubated twice on uncoated 10-cm culture dishes for 30 minutes to remove any remaining nonmyocytes, the nonattached viable cells were plated on gelatin-coated 24-well culture plates or 10-cm culture dishes and then cultured in DMEM (Life Technologies, Grand Island, NY) supplemented with 10% FCS (Life Technologies, Grand Island, NY). After 24-hour incubation in DMEM with FCS, the culture medium was changed to serum-free DMEM, and all experiments were performed 24 hours later. This purification procedure has well been established [11, 12], and >95% of the cells obtained by this method were cardiomyocytes.

### 2.2. Plasmid DNA

**Figure 1:**
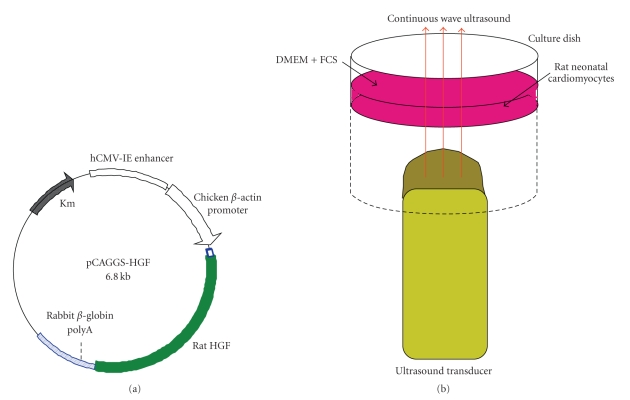
(a) Structure of the expression plasmid pCAGGS-HGF. The expression cassette of pCAGGS-HGF contains chicken *β*-actin promoter, rat HGF, and rabbit *β*-globin poly A. (b) Experimental setup. The transducer was attached to the bottom of the dish.

Preparation of rat hepatocyte growth factor (HGF) expression plasmid DNA was described previously [13]. Briefly, rat HGF cDNA cloned by polymerase chain reaction was inserted into the unique Xho I site between the cytomegalovirus immediate-early enhancer-chicken *β*-actin hybrid promoter and rabbit *β*-globin poly A site of the pCAGGS expression plasmid [14]. The resulting plasmid, pCAGGS-HGF, was grown in Escherichia coli DH5*α* (Figure 1(a)). The plasmid was purified with a QUIAGEN plasmid DNA kit and dissolved in TE buffer. The purified plasmid DNA was stored at −20°C and diluted to the required concentration with distilled water immediately before use.

### 2.3. Bubble Liposome

Liposome microbubble, SHU 508A, consists of palmitic acid and galactose and provides echogenic micron-sized air bubbles when suspended in water. The diameter of bubbles ranges from 2 to 8 *μ*m, and 97% are smaller than 6 *μ*m [15]. These air bubbles are stabilized by palmitic acid, which forms a molecular film that lowers the surface tension of the aqueous vehicle. The SHU 508A bubbles are nontoxic, have a neutral pH, are biodegradable, and are made from a physiologically occurring substance. The physiochemical properties of SHU 508A bubbles are typical of a saccharide solution [15].

### 2.4. Experiment on Ultrasound Mode

Before performing the experiments for dose-effect relationships using liposome sonoporation, we needed to determine the most appropriate ultrasound mode for the sonoporation procedure for efficient transfection. We tested four modes of ultrasound: pulsed wave Doppler, color flow Doppler, continuous wave Doppler, and harmonic power Doppler, which are available with standard echocardiographic machinery in a clinical laboratory. We performed a simple transfection experiment at the same acoustic pressure of 0.5 W/cm² for each ultrasound mode, using a single condition with 60 *μ*g HGF plasmid DNA, 1 × 10^7^ particles/mL of SHU 508A liposome, 30 sec insonification, 15 min of DNA incubation, and 3 repetitions of insonification. 

**Figure 2:**
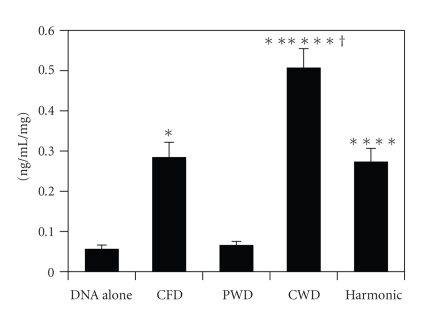
Comparison of four modes of ultrasound for sonoporation. Cells treated with continuous-wave Doppler ultrasound yielded the largest amount of HGF protein indicating this to be the most effective ultrasound mode. CFD: color flow Doppler; PWD: pulsed wave Doppler; CWD: continuous wave Doppler; Harmonic: harmonic power Doppler. *P < .05 versus DNA alone; **P < .05 versus CFD; ***P < .05 versus PWD; ^†^ *P* < .05 versus Harmonic.

Rat neonatal cardiomyocytes were inoculated and grown to confluence in DMEM+10% FCS. After confluence had been reached in a 35 mm Petri dish, the medium was changed to fresh defined serum-free medium. Plasmid DNA was diluted with distilled water immediately before the transfection. Each experiment was performed on 20 dishes. Cells on each dish were treated with ultrasound (Figure 1(b)). Pulsed wave Doppler, color flow Doppler, and continuous wave Doppler were insonified from PSK-25AT acoustic transducer with Toshiba SSA-380A (Toshiba Medical Systems), and harmonic power Doppler was insonified from S3 transducer with Sonos 5500 (Phillips Medical Systems). The experimental results are shown in Figure 2. Continuous-wave Doppler ultrasound was the most efficacious and was used for subsequent experiments.

### 2.5. Experiments for Dose-Effect Relations

The medium in 35 mm Petri dishes containing the cardiomyocytes was changed to fresh defined serum-free medium from DMEM+10% FCS. Rat HGF plasmid DNA was diluted with distilled water, and a volume corresponding to 60, 120, or 180 *μ*g was added to each of the 20 Petri dishes per DNA dose. Cells on each dish were then treated with continuous-wave Doppler ultrasound (frequency of 2.5 MHz and acoustic intensity of 0.5 W/cm² from a PSK-25AT acoustic transducer with Toshiba SSA-380A Ultrasound system) with SHU 508A liposome (1 × 10^7^ particles/mL) for acoustic exposure time of 30 or 60 seconds at room temperature (Figure 1(b)). In a separate series of experiments, we tested four liposome concentrations (0, 1 × 10^6^, 1 × 10^7^ or 1 × 10^8^ particles/mL), three insonification repetitions (1 insonification only, 3 or 5 insonifications for 30 seconds), and three DNA incubation times (15, 60 or 120 min). After the incubation, the culture medium was changed to normal DMEM+10% FCS and the cells were cultured for 72 hours. In a separate set of experiments, we examined the effect of culture period on the amount of DNA product that is HGF protein by discontinuing culture at 24, 48, and 72 hours and measuring the amount of rat HGF protein in the medium. The total amount of protein content in the cultured cells was measured and used to correct the HGF level in each dish. We measured rat HGF protein using an EIA kit (Institute of Immunology Co., Ltd., Tokyo, Japan) [13] and protein content of cultured cells using a Modified Lowry Protein Assay Kit (Pierce Biotechnology, Rockford).

### 2.6. Viability of Cultured Cells

To determine the safety of sonoporation, in a separate experiment, cultured cells were exposed to 0.1% trypan blue for 5 min just after ultrasound insonification. This allowed assessment of sarcolemmal membrane damage and was performed for each concentration of liposome, each insonification time, and each number of repetitions of insonification. The number of stained and unstained cells in the dishes was counted and used to calculate the percentage of intact cells [16]. The degree of cellular injury caused by sonoporation was determined by examining the insonified cells by scanning electron microscopy (Hitachi S-4800). Immediately after ultrasound insonification in the presence of liposome, the cardiomyocytes were fixed with phosphate-buffered 2.5% glutaraldehyde for 4 hours, followed by postfixation with 1% osmium tetroxide for 1 hour, and then were conventionally prepared for scanning electron microscopy.

### 2.7. Statistical Analysis

Data were expressed as the mean ± SEM. Comparisons of parameters from experimental groups were performed with unpaired *t* tests and resulting *P*-values were corrected according to the Bonferroni method. In analyses, *P* < .05 was considered to indicate statistical significance.

## 3. Results

### 3.1. Effect of Culture Period on HGF Protein Production by Sonoporated Cardiomyocytes

**Figure 3:**
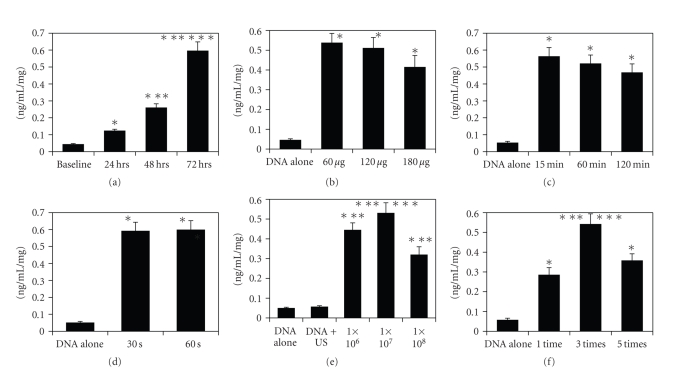
(a) Effect of culture period after transfection of HGF DNA on HGF protein production using 60 *μ*g of DNA and 1 × 10^7^ particles/mL liposome with three 30-sec insonifications and 15-min incubation with DNA. Baseline was the concentration of rat HGF protein in the culture medium around rat cardiomyocytes without any intervention at the beginning of cell culture. *P < .05 versus baseline; **P < .05 versus 24 hours after the onset of culture; ***P < .05 versus 48 hours after the onset of culture. (b) Effect of amount of plasmid DNA on HGF protein production using 1 × 10^7^ particles/mL liposome with three 30-sec insonifications and 15-min incubation with DNA. “DNA alone” indicates the concentration of rat HGF protein in the culture medium of cardiomyocytes treated with 60 *μ*g DNA without insonification. *P < .05 versus DNA alone. (c) Effect of incubation period of cardiomyocytes with plasmid DNA and liposome on HGF protein production using 60 *μ*g of DNA and 1 × 10^7^ particles/mL liposome with three 30-sec insonifications. *P < .05 versus DNA alone. (d) Effect of insonification time on protein production using 60 *μ*g of DNA, 1 × 10^7^ particles/mL liposome, and 15-min incubation with DNA, and three 30- or 60-sec insonifications. *P < .05 versus DNA alone. (e) Effect of liposome concentration on HGF protein production using 60 *μ*g of DNA with three 30-sec insonifications and 15-min incubation with DNA. *P < .05 versus DNA alone; **P < .05 versus 0 particles/mL; ***P < .05 versus 1 × 10^8^ particles/mL. (f) Effect of repetition of insonification on HGF protein production using 6 *μ*g of DNA and 1 × 10^7^ particles/mL liposome with 15-min incubation with DNA. *P < .05 versus DNA alone; **P < .05 versus 1 time; ***P < .05 versus 5 times.

The concentration of HGF protein in the culture medium increased as the culture period after ultrasonic transfection was extended. The transfection consisted of three 30-sec insonifications a 15-min incubation with HGF DNA (60 *μ*g) and liposome (1 × 10^7^ particle/mL) (Figure 3(a)). After 72 hours of culture, HGF protein concentration in the culture medium was measured and corrected using the protein content of the cultured cells.

### 3.2. Effect of the Amount of Plasmid DNA on HGF Protein Production by Sonoporated Cardiomyocytes

HGF protein concentration in the culture medium was 0.54 ± 0.049 ng/mL/mg and was highest when 60 *μ*g of DNA was administered with a liposome concentration of 1 × 10^7^ particles/mL, a15-min incubation, and three 30-sec insonification. Although the nominal mean values of HGF protein after transfection of 120 and 180 *μ*g DNA were lower than those after transfection of 60 *μ*g, the differences were not statistically significant (Figure 3(b)).

### 3.3. Effect of Incubation Period with Plasmid DNA and Liposome on HGF Protein Production by Sonoporated Cardiomyocytes

HGF protein concentration in the culture medium was 0.56 ± 0.053 ng/mL/mg and was highest when the incubation time was 15 min with a liposome concentration of 1 × 10^7^ particles/mL, 60 *μ*g DNA, and three 30-sec insonification. Although the mean values of HGF protein after transfection for 60 and 120 min were lower than those after 15 min incubation, the differences were not statistically significant (Figure 3(c)).

### 3.4. Effect of Insonification Time on HGF Protein Production by Sonoporated Cardiomyocytes

HGF protein concentration in the culture medium was 0.59 ± 0.052 ng/mL/mg and was highest when the insonification period was 30 sec with 60 *μ*g DNA, a liposome concentration of 1 × 10^7^ particles/mL, and 15-min incubation. There was no significant difference in HGF production in cells insonified for 30 and 60 min (Figure 3(d)).

### 3.5. Effect of Liposome Concentration on HGF Protein Production by Sonoporated Cardiomyocytes

HGF protein concentration in the culture medium was 0.53 ± 0.053 ng/mL/mg and was nominally highest when the liposome concentration was 1 × 10^7^ particles/mL and insonification consisted of three 30-sec ultrasound exposures, though it was statistically similar to that obtained with 1 × 10^6^ particles/mL. At a higher liposome concentration of 1 × 10^8^ particles/mL, HGF protein concentration decreased (Figure 3(e)).

### 3.6. Effect of Repetition of Insonification on HGF Protein Production by Sonoporated Cardiomyocytes

HGF protein concentration in the culture medium was 0.54 ± 0.053 ng/mL/mg and was highest when three 30-sec insonifications were given, with a liposome concentration of 1 × 10^7^ particles/mL and 60 mg DNA. This protein production was statistically higher than in cells given one or five insonifications (Figure 3(f)).

### 3.7. Effect of Insonification Time on Cell Viability

**Figure 4:**
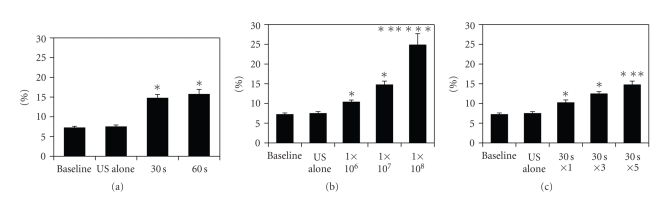
(a) Effect of insonification time on cell viability using 60 *μ*g of DNA, 1 × 10^7^ particles/mL liposome, and 15-min incubation with DNA, and three 30- or 60-sec insonifications. “US alone” represents the percentage of dead cells immediately after three 30-sec insonifications in the absence of liposome and DNA. *P < .05 versus baseline. (b) Effect of liposome concentration on cell viability using 60 *μ*g of DNA and three 30-sec insonifications and 15-min incubation with DNA. *P < .05 versus baseline; **P < .05 versus 1 × 10^6^ particles/mL; ***P < .05 versus 1 × 10^8^ particles/mL. (c) Effect of repetitions of insonification on cell viability using 60 *μ*g of DNA, 1 × 10^7^ particles/mL liposome, and 15-min incubation with DNA. *P < .05 versus baseline; **P < .05 versus 30 sec × 1.

The percentage of dead cells was 14.7 ± 0.9% and was higher in the cells given five 30-sec insonifications at a liposome concentration of 1 × 10^7^ particles/mL (Figure 4(a)). There was no statistical difference between 30- and 60-sec insonification.

### 3.8. Effect of Liposome Concentration on Cell Viability

The percentage of dead cells increased with increasing concentrations of liposome (Figure 4(b)). The dead cell count was 24.8 ± 2.9% and was highest when the liposome concentration was 1 × 10^8^ particles/mL and three 30-sec insonifications were used.

### 3.9. Effect of Number of Insonification Repetitions on Cell Viability

The percentage of dead cells increased as the number of insonification repetitions increased (Figure 4(c)). The dead cell count was 14.7 ± 0.9 % and was highest when five repetitions of the insonification step were given, with a liposome concentration of 1 × 10^7^ particles/mL.

### 3.10. Scanning Electron Microscopy Observations of Sonoporated Cardiomyocytes

**Figure 5:**
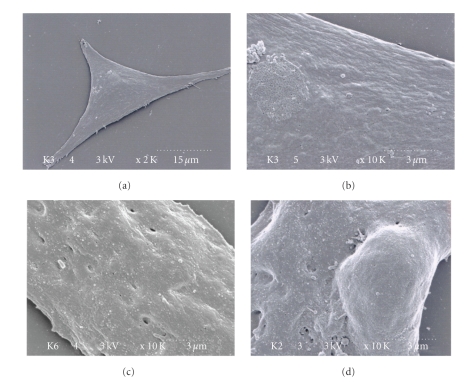
(a) and (b) Scanning electron microscopic images of intact cell surfaces of cultured cardiomyocytes. Scale dots are indicated on the images. (c) Image of a cell surface immediately after sonoporation using 1 × 10^6^ particles/mL liposome. (d) Image of a cell surface immediately after sonoporation using 1 × 10^8^ particles/mL liposome.

No particular changes were evident on the surfaces of untreated control cultured cardiomyocytes when viewed with the scanning electron microscope at low and high magnification (Figures 5(a) and 5(b)). After sonoporation with a low concentration of liposome (Figure 5(c)) and with a high concentration of liposome (Figure 5(d)), microdimples or pores were observed on the surfaces of the cultured cardiomyocytes.

## 4. Discussion

Considerable efforts have been made to develop methods that will allow effective and safe introduction of vectors into cells for gene therapy. However, we still need a breakthrough in the form of a novel vector that will transform cells at high efficiency and with low risk of adverse effects. This is especially true in cardiovascular medicine, where malignant cellular transformation is rare [17]. One of the promising candidates for safe and efficacious gene transfection is a naked plasmid vector that has been modified to have high affinity for cardiovascular tissues but which has no built-in viral components [17, 18]. We have developed a method for electroporation of a cytokine gene for treatment of cardiomyopathy [13]. However, using electric shock for transfection is not clinically practical. For this reason, we are pursuing the present sonoporation method as a protocol for gene transfection.

The HGF protein used in the present study is found in a wide variety of cell types and has multiple biological properties, including mitogenic, motogenic, morphogenic and antiapoptotic activities [19]. Several lines of evidence indicate that this molecule has potential for therapeutic use for treatment of heart failure, myocardial infarction, angina, and hypertension [20–22]. HGF may also have enormous therapeutic potential for hepatic and renal disorders, in addition to cardiovascular diseases [23–26]. 

In the present study, we showed variations in amount of HGF plasmid DNA, liposome concentration, the duration of insonification, and incubation time of the cardiomyocytes with liposome and DNA, and their dose relationships with the final amount of HGF protein released from the cultured neonatal cardiomyocytes. We found that specific amounts of liposome and repetitions of insonification were needed for effective protein production from cardiomyocytes. However, high concentrations of bubble liposome and large numbers of repeat insonifications resulted in decreased cell viability.

Plasma membrane sonoporation induced by ultrasound and subsequent self-sealing has been reported in previous investigations [27–29]. However, the exact mechanism by which membrane sonoporation causes substance incorporation into the cell is not yet understood. Some investigators speculate that the membrane poration results in both transfection efficiency and cellular damage. In the present study, scanning microscopy images revealed some microdimples or pores on the cell surface after sonoporation, which did not exist on the surface of control cardiomyocytes. The numbers of dimples or pores tended to increase with higher concentrations of liposome. Thus, we speculate that these dimples or pores on the cell surface might be related to transfection efficiency and might be evidence of cellular injury by sonoporation. Previous studies of sonoporation of vascular walls revealed that microbubble destruction would cause rupture of microvessels and extravasation [30–33], which would cancel out some benefits of sonoporation. Thus, the poration and self-sealing mechanism needs to be fully investigated and optimized. 

A sonoporation technique targeting the cardiovascular system has now been developed for gene transfection to myocardium, limb skeletal muscle, and arteries [34–37]. For a variety of target tissues, a number of microbubbles, including liposomes, and a range of ultrasound modes have been developed. The optimal combination of the type of microbubble, ultrasound mode, and target tissue still needs to be resolved [38–40]. However, the principal types of ultrasound used for sonoporation have included pulsed wave Doppler or continuous wave Doppler with acoustic pressure ranging 0.5–5 W/cm^2^ [34–37]. In the present study, we found that continuous wave Doppler at a standard frequency for clinical use, that is, 2.5 MHz and the usual acoustic pressure of 0.5 W/cm^2^, was most effective with our cardiomyocytes. The reason we used one of the standard ultrasound modes with standard settings for clinical use is that we would like to use our sonoporation system eventually in a clinical setting.

The present study has several limitations. To avoid the complexity of numerous combinations of experimental conditions, such as amount of DNA, concentration of liposome, duration of insonification, repeat count of insonification, length of incubation time, and culture period after gene transfection, we only used several practical combinations for an in vitro experiment for cultured cardiomyocytes. Thus, we might have missed other multimodal aspects of dose-effect relationships among these conditions.

## 5. Conclusion

HGF DNA was successfully transferred to cultured cardiomyocytes using sonoporation with a defined liposome concentration and a mode of insonification. A number of trade-offs between transfection efficiency and cellular injury have to be balanced to optimize this sonoporation method.
